# The Effect of a Casein and Gluten-Free Diet on the Epigenetic Characteristics of FoxP3 in Patients With Hashimoto's Thyroiditis

**DOI:** 10.7759/cureus.63208

**Published:** 2024-06-26

**Authors:** Elif S Aslan, Gulsen Meral, Ece Aydin, Sinan Caglayan, Aytug Altundag, Seyda Demirkol, Gizem Gormus, Mustafa Solak, Furkan Ayaz

**Affiliations:** 1 Molecular Biology and Genetics, Biruni University, Istanbul, TUR; 2 Molecular Biology and Genetics, Epigenetic Coaching, Norwich, GBR; 3 Endocrinology and Metabolic Diseases, Demiroğlu Bilim Üniversitesi, Istanbul, TUR; 4 Otorhinolaryngology, Biruni University, Istanbul, TUR; 5 Computer Science with Data Science, University of Sunderland, Sunderland, GBR; 6 Nutrition and Dietetics, Florence Nightingale Community Hospital, Istanbul, TUR; 7 Medical Genetics, Biruni University, Istanbul, TUR

**Keywords:** thyroid, insulin resistance, hashimoto's thyroiditis, methylation, foxp3

## Abstract

Background

Hashimoto's thyroiditis (HT) is an autoimmune thyroid disease characterized by inflammation and dysfunction of the thyroid gland, resulting in hypothyroidism, it results in impaired thyroid hormone generation and mimics hypothyroidism. The disease involves complex interactions among genetic, environmental, and epigenetic factors, particularly affecting the regulation of T regulatory (Treg) cells, including CD4 + *foxp3 *+ T cells. Treg cells, defined as CD4 + T cells, rely on the expression of the *foxp3 *transcription factor, which is crucial for their development and differentiation. Disruptions in this regulation can lead to immune dysregulation and potential proinflammatory responses. The study focuses on investigating the impact of dietary patterns on the epigenetic changes in the *foxp3* gene, a key player in the development of HT. The primary aim was to evaluate how eliminating gluten and casein proteins from dietary regimens may influence the methylation levels of the *foxp3* gene, considering the potential link between these dietary components and the triggering of autoimmune diseases.

Methods

An epigenetic analysis of the *foxp3* gene in HT patients who were strictly following a dietary plan compared with the control group. For the epigenetic study, a methylation analysis experiment was conducted.

Results

Our findings revealed a notable reduction in *foxp3* gene methylation levels among HT patients who adhered to a diet excluding casein and gluten. The control maintained normal dietary guidelines and showed no significant alterations in methylation levels.

Discussion

The laboratory values showed a decrease in methylation levels of the *foxp3* gene, with statistical significance indicated as *p<0.005, **p<0.001, ***p<0.0001, suggesting a potential enhancement in its expression which could have profound implications for immune system regulation. Disruptions in the *foxp3* pathway are crucial in the development of autoimmune disorders, where altered activity hinders the regulation of T cell (Treg) development, ultimately contributing to conditions like HT disease. These findings imply that nutritional interventions, especially for individuals with HT, could potentially be a strategy for mitigating autoimmunity through epigenetic mechanisms.

## Introduction

Hashimoto's thyroiditis (HT) is an autoimmune thyroid disease characterized by inflammation of the thyroid gland, typically presenting with symptoms of hypothyroidism. The disease involves the loss of immune tolerance to thyroid autoantigens [1]. Autoantibodies, such as thyroglobulin (TG) and thyroid peroxidase (TPO), are commonly detected in patients [2,3]. The exact causes of autoimmune thyroid diseases remain unclear, but it is known that genetic and environmental factors, along with epigenetic changes and immune regulation alterations, play significant roles [4,5].

Environmental factors, including diet, stress, and infections, can influence these epigenetic modifications, such as DNA methylation and histone modifications. These modifications can alter *foxp3* expression, a crucial gene in immune regulation. DNA methylation and histone modifications are key epigenetic mechanisms that regulate gene expression, affecting the physical structure of the genome and determining gene activity across different cell types and conditions. Abnormalities in these processes can lead to various diseases [4-6]. For example, increased methylation in the *foxp3 *gene's promoter region can decrease its expression, impairing the function of regulatory T cells (Tregs). Tregs, marked by the presence of CD4 and *foxp3*, are critical for maintaining immune tolerance by ensuring the immune response does not target the body's own tissues [4-6].

Cross-reactivity between thyroid peroxidase (TPO) and lactoperoxidase (LPO) due to their structural similarities can also contribute to thyroid autoimmunity. The immune system may mistakenly target thyroid cells containing TPO when encountering LPO in various tissues [3]. The transcription of *foxp3* is influenced by signals from T cell receptors (TCR), various cytokines, and epigenetic factors. The balance between the innate and adaptive components of the immune system is crucial, with Tregs and *foxp3* playing vital roles in maintaining this balance and ensuring immune homeostasis. Single nucleotide polymorphisms (SNPs) and epigenetic modifications, such as changes in DNA methylation and histone acetylation patterns, contribute to the pathogenesis of immune dysregulation in HT [6-8].

Histone acetylation involves the addition of an acetyl group to the lysine residues of histone proteins wrapped around DNA, typically associated with transcriptional activation and increased gene expression. This process is carried out by enzymes known as histone acetyltransferases (HATs) and is crucial for the development and maintenance of immune tolerance and the expression of the *foxp3* transcription factor [9-11].

The recognition of *foxp3* as a key regulator of Treg cell development and function has provided important insights into the biology of Treg cells in the context of disease development and control [12-14]. Alongside transcriptional and post-translational regulations, the formation of a specific epigenetic signature during the development phase is critical for the proper functioning of Treg cells [11]. Changes in *foxp3* expression or shifts in these epigenetic patterns can contribute to the instability and aberrant behavior of Treg cells in autoimmune diseases. In a healthy state, the expression of *foxp3* is carefully regulated through transcriptional control, epigenetic regulation, post-translational modifications, microRNAs, and other factors [11,12]. The overall epigenetic signature is maintained by histone acetyltransferases (HATs), histone deacetylases (HDACs), DNA methylation, and other factors [12,13,15]. In individuals with a genetic predisposition, environmental factors can trigger inflammation, leading to a subset of Tregs losing *foxp3 *expression, a phenomenon known as ex-*foxp3*, rendering them unstable [16,17].

Several environmental factors, such as diet, stress, and smoking, have been hypothesized as potential contributors to autoimmunity as a multifactorial disorder. Undoubtedly, dietary habits are significant risk factors for developing autoimmune diseases [18]. Although there is no specific diet for treating autoimmune diseases, certain nutrients can influence the immune system's function. These nutrients can help prevent inflammation by reducing the production of pro-inflammatory cytokines and enhancing regulatory T-cell function [19]. However, the effectiveness and significance of a gluten-free and casein-free diet for autoimmune diseases remain controversial. Some studies suggest that such a diet can help ameliorate various symptoms, including those related to social interactions, cognition, communication, stereotypical behaviors, attention, and emotion [20]. A gluten-free diet involves eliminating grains that contain gluten, such as wheat, rye, barley, and potentially oats. On the other hand, a casein-free diet requires the removal of mammalian milk and its products, including butter and cheese [20,21].

The primary aim of our study is to explore the epigenetic modifications in the *foxp3* gene that are pivotal in the development of HT in response to dietary interventions. We focus on the impact of diets that exclude casein and gluten, which have been implicated in significant epigenetic alterations associated with autoimmune diseases. By assessing the methylation levels of the *foxp3* gene before and after implementing a gluten-free and casein-free diet, our research aims to understand how eliminating these dietary components influences the epigenetic landscape and potentially mitigates the progression of autoimmune diseases. A dairy-free diet model, which excludes all dairy products and derivatives, ensures that no sources of casein and gluten are consumed.

## Materials and methods

Methods of pilot study

The Ethics Committee of Biruni University approved the current study protocol numbered 2015-KAEK-47- 21-01; 2021-06.

Subjects

The clinical data of patients diagnosed with HT were collected from the University of Biruni Hospital. The eligibility criteria for the study were the following: participants had to be adults aged 18 or older with laboratory-confirmed HT, indicated by positive biomarkers of anti-TPO and/or anti-Tg antibodies. Additionally, they needed to have a body mass index (BMI) over 30, no chronic medication use or history of chronic illnesses, and not be receiving any treatment other than for HT. Exclusion criteria encompassed pregnancy, being pregnant during follow-up, discontinuation of follow-up and treatment, and cessation of the gluten-free and casein-free diet protocol prepared according to international standardization. For a duration of three months, the dietary patterns of 20 patients with HT and insulin resistance, as well as 20 control patients, were prospectively monitored and supervised by an expert dietitian and endocrinology and metabolism diseases specialist. Blood samples have been collected from patients with HT and insulin-resistant patients stored in ethylenediaminetetraacetic acid (EDTA) tubes with purple caps and subjected to DNA isolations. Following blood collection, the samples have been preserved at +4 degrees Celsius prior to DNA isolation. This study was completed without altering any conditions.

DNA isolation

DNA was isolated from whole blood samples by using the Quick-DNA Miniprep Plus Kit (D4068; Zymo Research, Irvine, California). Elution was carried out using 50 µl of (deoxyribonuclease/ribonuclease) DNAse/RNAse-free water to the samples isolated by following the biological fluids section of the kit. The DNA amounts of the samples were measured after isolation. The concentration of each sample was normalized as 4 ng/µl. This normalization was carried out for the next step.

The analysis of one-step qMethyl

The one-step percent methylation analysis experiment was carried out using the OneStep qMethyl-PCR Kit (D5310; Zymo Research, Irvine, California). Five µl (20 ng) of isolated DNA samples were added to 10 µl of the test or reference mix, 2 µl of primer mix, and 3 µl of DNAse/RNAse-free water according to the protocol of kit. The samples were loaded to the Mic real-time polymerase chain reaction (qPCR) device (Bio Molecular Systems, Queensland, Australia) with methylated and unmethylated DNA standards. Since the total number of samples was 44, the experiment was carried out with a total of two runs. Methylated and unmethylated standards were loaded in both runs. The experimental protocol is as follows in Table 1.

**Table 1:** The experimental protocol of PCR for 40 cycles MSRE - methylation-sensitive restriction enzyme; PCR - polymerase chain reaction

Steps	Temperature	Time
MSRE digestion	37°C	2 hours
Initial denaturation	95°C	10 minutes
Denaturation	95°C	30 seconds
Annealing	54°C	60 seconds
Extension	72°C	60 seconds
Final extension	72°C	7 minutes

Percent methylation calculation with dCt

After the test results, data were transferred to Excel (Microsoft, Redmond, Washington); the DCt value was calculated by subtracting the reference value from the test value for percent methylation calculation. Then, this value was calculated using the percentage methylation formula 2(-DCt)*100 to calculate the percentage methylation value of the samples. In terms of visual presentation, tables of these results were created for two separate genes (foxp3). The primer design of the* foxp3* is shown in Table 2.

**Table 2:** Primer design of foxp3 gene UCSC - University of California, Santa Cruz; PCR - polymerase chain reaction; HPLC - high-performance liquid chromatography

Primer sequences	Length
*Foxp3 *F: GCCCCCGACT…	-
GCC CCC GAC TTG CCC AGA TT	20 bp HPLC
*Foxp3 *R: GGACAGGGCA…	-
GGA CAG GGC AGC CAG TTC TCG	21 bp HPLC
Primers are for *foxp3*; checked on UCSC in-silico PCR	-

## Results

This study investigated the effects of a diet excluding casein (a protein found in milk) and gluten (a protein found in wheat) on the methylation levels of the *foxp3* gene in patients with HT. The results indicated a significant reduction in the methylation levels of the *foxp3* gene in patients who adhered to the casein-free and gluten-free diet. In contrast, control groups comprised of healthy individuals not following any specific diet did not show significant changes in the methylation levels of the *foxp3* gene, suggesting that the changes observed in the HT patients were specifically due to the dietary intervention.

**Figure 1:**
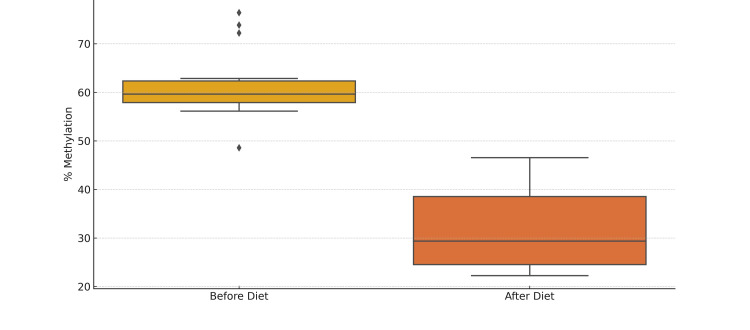
Methylation levels before and after diet in the experimental group This box plot illustrates the changes in *foxp3* gene methylation levels in HT disease patients before and after dietary adjustments. The boxes depict the median, the first and third quartiles, and the overall data range. This box plot illustrates the changes in *foxp3* gene methylation levels in HT disease patients before and after dietary adjustments. The boxes depict the median, the first and third quartiles, and the overall data range. Prior to the diet, the median methylation level was observed at 45.47%, with a mean of 45.30% and a standard deviation of 5.04%. These values indicate the central tendency and variability of methylation levels among the subjects before dietary changes. Following the diet, the median methylation level increased to 47.10%, and the mean rose to 47.33%. This increase in both the median and mean values suggests a shift towards higher methylation levels post-diet. Additionally, the standard deviation decreased to 4.14%, indicating a reduction in the variability of methylation levels among the subjects after the diet. The plot highlights a significant decrease in methylation levels following the dietary changes. The plot highlights a significant decrease in methylation levels following the dietary changes.

**Figure 2:**
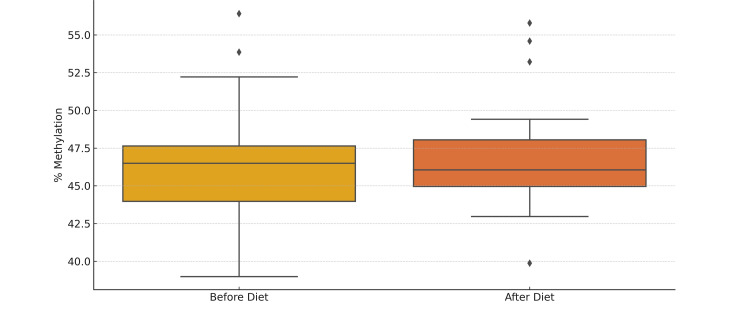
Methylation levels before and after diet in the control group This box plot illustrates the distribution of *foxp3 *gene methylation levels in the control group before and after dietary changes. Each box represents the median, the first and third quartiles, and the overall range of the data. The plot shows that methylation levels remain relatively stable in the control group.

**Figure 3:**
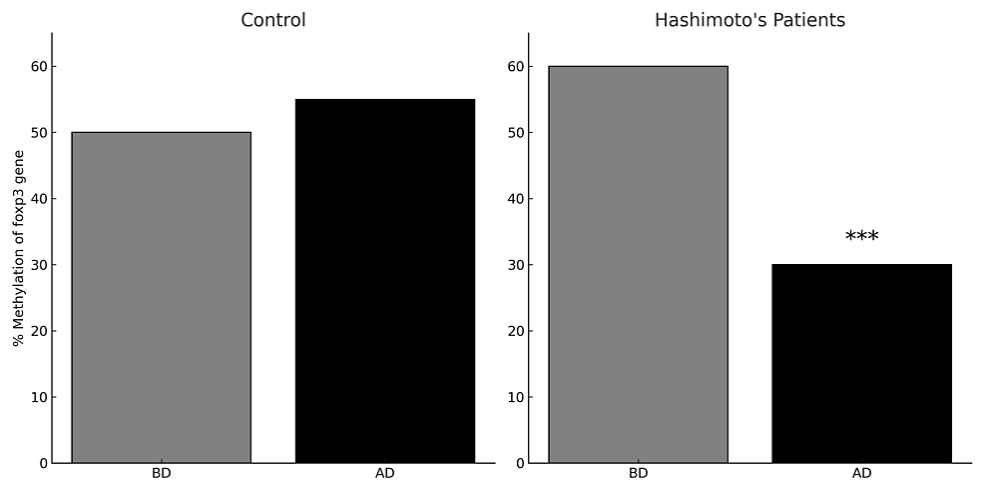
Methylation levels (%) of foxp3 gene in the Hashimoto's disease patients and control groups before and after the dietary changes Methylation levels (%) of *foxp3* gene in the Hashimoto's disease patients and control groups before and after the dietary changes. Y-axis - methylation levels (%); X-axis - groups (Hashimoto's disease patients before diet (BD), Hashimoto's disease patients after diet (AD), control groups BD, control groups AD; the Student t-test was applied to the groups to draw the statistical significance as, *p < 0.005, **p < 0.001, ***p < 0.0001. Before the diet, the median methylation level was 45.47%, with a mean of 45.30% and a standard deviation of 5.04%, reflecting the initial spread of methylation levels among subjects. After the diet, the median methylation level rose to 47.10% and the mean increased to 47.33%, indicating a shift towards higher methylation levels. Furthermore, the standard deviation decreased to 4.14%, suggesting a reduction in variability among subjects post-diet. The figure includes data points representing the percentage of methylation of the *foxp3* gene in both Hashimoto's disease patients and control groups, both before and after dietary changes.

The study monitored dietary patterns over three months for 20 patients with HT (Figure 1) and insulin resistance, as well as 20 healthy control individuals (Figure 2). Methylation levels of the *foxp3* gene were analyzed in both groups. Statistical analysis showed that while the healthy control group did not exhibit significant changes in *foxp3 *methylation levels post-diet, the HT patients experienced a substantial and significant decrease in *foxp3* methylation levels post-diet compared to pre-diet levels (Figure 3). These findings suggest that the diet may reduce *foxp3* gene methylation, potentially enhancing its expression.

The analysis of methylation levels in the control group, both before and after the diet intervention, demonstrates significant statistical changes that support the impact of dietary modifications on epigenetic markers. Before the dietary changes, the median methylation level among the subjects was recorded at 45.47%, with a mean of 45.30% and a standard deviation of 5.04%. These values highlight the central tendency and variability of methylation levels within the group. After the dietary intervention, there was an increase observed in these values: the median methylation level rose to 47.10%, and the mean increased to 47.33%. This shift indicates an overall increase in methylation levels following the diet. Additionally, the standard deviation dropped to 4.14%, showing that the variability in methylation levels among the subjects decreased after the diet. This means that the methylation levels became more uniform across the group following the dietary changes, suggesting a more consistent response to the intervention among all participants.

The combination of an increase in central tendency measures (median and mean) and a decrease in variability (standard deviation) provides robust evidence that the diet had a stabilizing effect on methylation levels, leading to higher and more uniform methylation across the group. These findings are critical in understanding the role of diet in modulating epigenetic markers and can inform future research and dietary guidelines aimed at improving epigenetic health outcomes.

 As a result; the reduction in methylation levels of the *foxp3* gene in HT could potentially enhance the expression of *foxp3* and increased *foxp3* expression can improve the function of Tregs, which are critical for maintaining immune tolerance and preventing the immune system from attacking the body's own tissues. The reduction in methylation levels of the *foxp3* gene in HT enhances *foxp3* expression. Increased *foxp3* expression improves the function of Tregs, which are crucial for maintaining immune tolerance. Enhanced Treg function helps prevent the immune system from attacking the body's own tissues, thereby mitigating autoimmune responses and promoting overall immune system balance. This understanding underpins the potential use of dietary or pharmacological interventions to modulate gene methylation and improve immune regulation in autoimmune diseases.

## Discussion

In our study, we focused on the patients with HT. The co-occurrence of multiple autoimmune diseases with autoimmune thyroiditis suggests that these conditions may be induced by environmental factors, emphasizing the importance of epigenetic mechanisms through which environmental factors can trigger autoimmunity [22]. Several single nucleotide polymorphisms (SNPs) have been investigated for their potential associations with HT disease. For instance, a meta-analysis identified a possible link between HT and the *foxp3*-3279 SNP. Epigenetics offers a potential connection between genetics, environmental factors, and the HT phenotype [23]. Studies have also shown that increased methylation of FOXK2, a member of the forkhead box (FOX) family, is associated with higher thyroid-stimulating hormone (TSH) concentrations [24,25]. The forkhead box (FOX) gene family encodes proteins that regulate the transcription of genes participating in a number of functions, including the development of various organs, regulation of senescence or proliferation, and metabolic homeostasis [25]. The findings suggest that dietary interventions, specifically the exclusion of casein and gluten, may serve as a strategy to modulate immune function in HT patients through epigenetic mechanisms by lowering the methylation levels of the *foxp3* gene.

The results indicate a multifactorial impact involving both genetic and environmental factors because diseases or conditions influenced by both genetic and environmental factors are described as multifactorial. Genetic variants constitute the primary risk, encompassing both immune regulatory genes and target tissue genes related to autoimmune thyroid sensitivity [26, 27]. This primary genetic risk can interact with environmental factors such as infection, diet, and iodine exposure, creating a synergistic effect that may trigger the disease [26]. While genetic predispositions lay the groundwork for potential autoimmune disorders, environmental factors often play a critical role in sparking the actual onset of the disease. Elements like stress, infections, and exposure to specific chemicals or foods can interact with our genetic vulnerabilities, triggering or worsening thyroid autoimmunity. By understanding how our genes influence these conditions, we can better predict who might be at risk, tailor treatment plans more effectively, and possibly develop ways to prevent the disease from developing in the first place. The key to genetic-environmental interactions lies in epigenetic modulation. Identifying the underlying genetic-epigenetic interactions in autoimmune thyroid conditions will pave the way for promising therapeutic targets [26,27,5]. The term "epigenetic modulation" refers to changes in gene expression that don't involve alterations to the underlying DNA sequence but are still influenced by genetic and environmental factors. This concept is critical in understanding how various external factors like diet, stress, and exposure to toxins can impact gene activity, potentially affecting health and disease states without changing the DNA itself [5].

In autoimmune and allergic conditions, significant epigenetic mechanisms in *foxp3* and Tregs have been observed in studies related to nutrition. The link between nutrition, epigenetic modifications in *foxp3*, and the function of Tregs is a crucial area of research that has significant implications for understanding and managing autoimmune diseases. By influencing these epigenetic mechanisms, we can potentially direct immune responses in a way that prevents or mitigates autoimmune and allergic conditions [12]. For instance, during cow's milk allergy (CMA), changes in DNA methylation of Th1 and Th2 cytokine genes were observed, and after treatment with extensively hydrolyzed casein formula (EHCF) + *Lactobacillus rhamnosus *GG (LGG), patients who gained oral tolerance showed significant demethylation of the *foxp3* TSDR gene compared to children treated with other formulas [12,26,28-30]. Enhancing the regulatory function of Tregs through dietary changes could potentially lead to better management of HT [29,30]. This approach highlights the role of nutrition in influencing genetic expression and its potential therapeutic benefits in autoimmune disorders [19,20,23].

Forkhead box transcription factor 3 (*foxp3*) is essential for the development and function of regulatory T cells (Tregs) and plays a role in acquiring oral tolerance. In children with IgE-mediated CMA, the acquisition of tolerance has been associated with the demethylation of *foxp3*'s Treg-specific demethylated region (TSDR) [31, 27]. Other studies have shown that gluten can impact the microbiome, increase intestinal permeability, and influence oxidative stress, potentially affecting epigenetic mechanisms. Gluten can affect individuals with gluten sensitivity by triggering an immune response that leads to inflammation in the gut. This inflammatory response can disrupt the gut barrier, leading to increased intestinal permeability (leaky gut) [9]. In individuals sensitive to gluten, consuming it can lead to inflammation in the gut. This inflammation can interfere with the functioning of regulatory T cells (Tregs), which are crucial for controlling immune responses. The ongoing inflammation might weaken the effectiveness of *foxp3*, a key factor that helps Tregs work properly. When *foxp3* isn't working well, Tregs can't regulate the immune system effectively, potentially leading to an overactive immune response. This not only worsens inflammation in the gut but could also impact other areas, such as the thyroid, contributing to further health issues [4,15].

Our study supports these findings, demonstrating that a gluten-free diet can similarly benefit epigenetic behavior and immune function in patients with HT [9,32]. Substance P is a chemical in the body known for triggering inflammation. It activates immune cells, prompting them to release inflammatory substances called cytokines. The connection between substance P and gluten sensitivity is an area that merits further research to clarify its exact role and potential as a target for managing the symptoms and inflammatory aspects of gluten-related disorders [20-22,33].

The study underscores the significance of dietary interventions in managing autoimmune diseases through epigenetic mechanisms. By excluding casein and gluten, HT patients may experience a beneficial reduction in the methylation of the *foxp3* gene, potentially enhancing immune regulation and providing a novel strategy for managing autoimmunity. While the idea of eliminating certain foods like gluten and casein from the diet is not new, applying this strategy specifically for influencing epigenetic changes, such as those affecting the *foxp3* gene involved in immune regulation, presents a fresh perspective. This approach focuses on managing autoimmune diseases like HT by targeting the underlying genetic mechanisms, not just alleviating symptoms. This nuanced application could reshape how we understand and manage these conditions, making it a promising area for further exploration in medical treatment.

Our research findings show that a diet free from casein and gluten significantly affects the methylation of the *foxp3* gene in patients with HT. Specifically, a gluten-free diet excludes grains like wheat, rye, barley, and sometimes oats, which contain gluten. A casein-free diet, on the other hand, involves avoiding mammalian milk and dairy products like butter and cheese. Prior to the diet, the median methylation level was observed at 45.47%, with a mean of 45.30% and a standard deviation of 5.04%. These values indicate the central tendency and variability of methylation levels among the subjects before dietary changes. Following the diet, the median methylation level increased to 47.10%, and the mean rose to 47.33%. This increase in both the median and mean values suggests a shift towards higher methylation levels post-diet. Additionally, the standard deviation decreased to 4.14%, indicating a reduction in the variability of methylation levels among the subjects after the diet.

These results are crucial for comprehending how diet influences epigenetic markers. They pave the way for future studies and dietary recommendations focused on enhancing epigenetic health outcomes. These results suggest that nutritional interventions, particularly in HT patients, may serve as a potential strategy for managing autoimmunity through epigenetic mechanisms [15,32].

As a result, the application of a casein-free and gluten-free diet in HT patients was found to lead to a decrease in the methylation level of the *foxp3* gene. This study significantly advances the field of epigenetic research by investigating how these dietary changes influence the methylation levels of the *foxp3* gene, thereby affecting genetic expression. The use of thyroid-stimulating hormone (TSH) levels and the functionality of regulatory T cells (Tregs) as biomarkers offers direct measures of thyroid function and immune regulation, directly tying into the disease's pathology. Furthermore, the study's innovative personalized approach, which intertwines genetics, nutrition, and epigenetics, exemplifies a multidisciplinary strategy that could lead to novel therapeutic options for autoimmune diseases.

The study also faces several limitations that could impact the validity and applicability of its findings. The small sample size may limit the generalizability of the results, potentially skewing data interpretation due to statistical constraints. Additionally, the short duration of the dietary intervention may not fully capture the long-term effects essential for understanding chronic autoimmune conditions. A lack of diversity in the participant pool could prevent the findings from being applicable to all demographics affected by HT. Furthermore, the absence of strict control and monitoring of dietary compliance could introduce variability that undermines the reliability of the link between diet and epigenetic changes. Lastly, uncontrolled confounding factors such as physical activity, stress levels, and environmental exposures could also distort the results, complicating the interpretation of the diet's true impact on autoimmune disease.

## Conclusions

These findings may help us understand the effects of a casein-free and gluten-free diet on the* foxp3* gene in HT patients. Further research is needed to develop nutrition-focused, personalized interventions as a potential strategy for managing autoimmune diseases and metabolic health. The limitations of the study include a small sample size and the potential masking effect of genetic factors on dietary effects. Larger-scale, long-term clinical studies are required to validate these findings. Our study demonstrates that a casein-free and gluten-free diet significantly reduces *foxp3* gene methylation in patients with HT, potentially enhancing immune regulation through improved function of regulatory T cells (Tregs). In contrast, healthy controls showed no significant changes in methylation levels, highlighting the diet's specific benefits for HT patients. These findings align with other research showing the positive impact of dietary interventions on epigenetic mechanisms and immune function in autoimmune conditions. The study suggests that genetic and environmental factors, particularly diet, play a crucial role in autoimmune thyroid diseases. This research supports the development of personalized dietary strategies for managing autoimmune diseases through epigenetic modulation. In conclusion, a casein-free and gluten-free diet shows promise as a novel strategy for improving immune regulation and managing HT, highlighting the important role of nutrition in autoimmune disease management.
